# Aspirin and 5-Aminoimidazole-4-carboxamide Riboside Attenuate Bovine Ephemeral Fever Virus Replication by Inhibiting BEFV-Induced Autophagy

**DOI:** 10.3389/fimmu.2020.556838

**Published:** 2020-11-24

**Authors:** Hsu-Hung Tseng, Wei-Ru Huang, Ching-Yuan Cheng, Hung-Chuan Chiu, Tsai-Ling Liao, Brent L. Nielsen, Hung-Jen Liu

**Affiliations:** ^1^ Institute of Molecular Biology, National Chung Hsing University, Taichung, Taiwan; ^2^ Division of General Surgery, Taichung Hospital, Ministry of Health and Welfare, Taichung, Taiwan; ^3^ The iEGG and Animal Biotechnology Center, National Chung Hsing University, Taichung, Taiwan; ^4^ Department of Medical Research, Taichung Veterans General Hospital, Taichung, Taiwan; ^5^ Rong Hsing Research Center for Translational Medicine, National Chung Hsing University, Taichung, Taiwan; ^6^ Ph.D Program in Translational Medicine, National Chung Hsing University, Taichung, Taiwan; ^7^ Department of Microbiology and Molecular Biology, Brigham Young University, Provo, UT, United States; ^8^ Department of Life Sciences, National Chung Hsing University, Taichung, Taiwan

**Keywords:** 5-aminoimidazole-4-carboxamide-1-β-riboside, aspirin, PI3K/Akt/NF-kB, autophagy, bovine ephemeral fever virus

## Abstract

Recent study in our laboratory has demonstrated that BEFV-induced autophagy *via* activation of the PI3K/Akt/NF-κB and Src/JNK pathways and suppression of the PI3K-AKt-mTORC1 pathway is beneficial for virus replication. In the current study, we found that both aspirin and 5-aminoimidazole-4-carboxamide-1-β-riboside (AICAR) siginificantly attenuated virus replication by inhibiting BEFV-induced autophagy *via* suppressing the BEFV-activated PI3K/Akt/NF-κB and Src/JNK pathways as well as inducing reversion of the BEFV-suppressed PI3K-Akt-mTORC1 pathway. AICAR reversed the BEFV-activated PI3K/Akt/NF-κB and Src/JNK pathways at the early to late stages of infection and induced reversion of the BEFV-suppressed PI3K-AKt-mTORC1 pathway at the late stage of infection. Our findings reveal that inhibition of BEFV-induced autophagy by AICAR is independent of AMPK. Furthermore, we found that AICAR transcriptionally downregulates the ATG related genes ULK1, Beclin 1, and LC3 and enhances Atg7 degradation by the proteasome pathway. Aspirin suppresses virus replication by inhibiting BEFV-induced autophagy. It directly suppressed the NF-κB pathway and reversed the BEFV-activated Src/JNK pathway at the early stage of infection and reversed the BEFV-suppressed PI3K/Akt/mTOR pathway at the late stage of infection. The current study provides mechanistic insights into the effects of aspirin and AICAR on BEFV replication through suppression of BEFV-induced autophagy.

## Introduction

Bovine ephemeral fever (BEF) is an acute febrile illness of cattle and water buffalo. Clinically, it is characterized by sudden onset of fever, stiffness, depression, nasal discharges, joint pain, and lameness in three days (1). Although the mortality rate is low, there are serious economic impacts including loss of milk production in widespread regions of Africa, the Middle East, Asia, and Australia (2). To date, treatment of BEF is mainly supportive and symptomatic. BEFV is a negative, single stranded RNA virus belonging to the rhabdovirus family in the order of Mononegavirales. BEFV virions are cone shaped (3), composed of a single-stranded, negative-sense RNA genome with a lipid envelope and five structural proteins, (L, P, G, N, and M) (4). The matrix (M) protein of BEFV is a nucleocytoplasmic shuttling protein (5) and is essential for virus maturation, budding, and regulation of the expression of viral and host proteins (6). It also plays an important role in inducing autophagy during BEFV infection (7). The PI3K/Akt signaling pathway and its downstream target, the mammalian target of rapamycin (mTOR), are involved in the regulation of diverse cellular functions. Many viruses target and hijack this pathway for host cell entry or viral protein translation (8, 9), especially for RNA synthesis of non-segmented, negative-stranded RNA viruses (9). An earlier study has shown that inhibition of PI3K and mTOR has positive effects on BEFV replication (10). Cell entry of BEFV follows a clathrin-mediated and dynamin 2-dependent endocytosis pathway that requires Rab5 and Rab7 as well as microtubules (11). In addition, upregulation of the PI3K-Akt-NF-κB and Src-JNK-AP1 pathways by BEFV are essential not only for cell entry (12) but also trigger autophagy for virus replication (7). During cell entry, BEFV also triggers cyclooxygenase-2 (Cox-2)-catalyzed prostaglandin E2 (PGE2) synthesis and induces expressions of G-protein-coupled E-prostanoid (EP) receptors 2 and 4, leading to amplification of these pathways (12). Aspirin, acetylsalicylic acid, is one of the commonly used non-steroidal anti-inflammatory drugs (NSAID) for analgesic, antipyretic, and anti-inflammatory therapy. It causes an irreversible inactivation of Cox-1 and Cox-2 and sequentially inhibits the formation of PGE2, thus reducing the inflammation reaction (13). This infers an inhibitory role of aspirin to BEFV.

Autophagy can be induced by interplays between AMP-activated protein kinase (AMPK), mTOR, and Unc-51 like autophagy activating kinase (ULK 1/2) (14). It was reported that mTOR serves as a main gate way to autophagy under amino acid stimulation (15). mTOR complex 1 (mTORC1) is a repressor of autophagy under nutrient sufficiency conditions (16). In energy critical situations, AMPK induces autophagy by activating ULK 1/2 and by suppressing mTORC1 through activating tuberous sclerosis complex 2 (TSC2) or inhibiting the regulatory-associated protein of mTOR (raptor) (17, 18). Our recent study has shown that BEFV induces autophagy by suppression of mTORC1 (7). It is interesting to examine if AMPK is involved in BEFV-induced autophagy. AMPK can be activated indirectly by a modulator that causes AMP or calcium accumulation or directly binds to and activates AMPK by conformational changes (19). 5-aminoimidazole-4-carboxamide riboside (AICAR) is a common AMPK activator; it is metabolized to AICAR 5’-monophosphate (ZMP) to bind to the AMPKγ subunit without changing the ADP : ATP ratio or altering oxygen uptake (20). Interestingly, our findings reveal that AICAR inhibits BEFV-induced autophagy in an AMPK-independent mechanism. Collectivelly, the study provides mechanistic insights into aspirin- and AICAR-modulated inhibition of BEFV-induced autophagy *via* suppressing the BEFV-activated PI3K/Akt/NF-κBand Src/JNK pathways as well as reversion of BEFV-inactivated PI3K/Akt/mTORC1, thereby inhibiting virus replication.

## Materials and Methods

### Virus Titration

Madin-Darby bovine kidney (MDBK) cells were infected with BEFV for 24 h. The supernatant containing BEFV particles was collected and serially diluted with serum-free DMEM. Each serial diluted virus solution (200 μl) was seeded in a 24-well-plate to incubate with the MDBK cells for 1 h. Unabsorbed viruses were removed by washing the cells with phosphate buffered saline (PBS). Then, the cells were overlaid with DMEM containing 2% FBS and 0.6 ml of 0.8% agarose. After incubation at 37°C for 2 to 3 days. BEFV formed plaques staining by neutral red for 3 h were counted.

### Cells and Viruses

MDBK cells were cultured in Dulbecco’s modified eagle medium (DMEM) supplemented with 10% fetal bovine serum (FBS). (1x10^6^) cells were seeded in 6-cm cell culture dishes one day before initiating the experiment and were incubated at 37 °C with 5% CO_2_. The 2004/TW/TN1 strain of BEFV was propagated in MDBK cells. The supernatants of BEFV-infected cells were harvested when 70%–80% cytopathic effect (CPE) was detected, and then concentrated by Polyethylene glycol (PEG) 6000 precipitation. The harvested BEF viruses were dialysed and resuspended in phosphate-buffered saline (PBS), then stored at -70°C before use.

### Chemical Inhibitors and Reagents

5-aminoimidazole-4-carboxamide-1-β-riboside (AICAR) and Furancarboxylic acid were purchased from Calbiochem Co. (San Diego, USA). Aspirin, indomethacin, MG132, and NS-398 (Cox-2 specific inhibitor) were purchased from Sigma-Aldrich Co. Prostaglandin E2 (PGE2) EIA kit was purchased from Cayman Chemical Co. (Ann Arbor, USA).

### Antibodies

The catalog numbers and dilution factor of the primary antibodies antibodies used in this study are shown in **Table 1**. Polyclonal antibodies against the BEFV M protein are from our laboratory stock. Anti-rabbit IgG (H + L) and anti-mouse IgG (H + L) antibodies were purchased from Kirkegaard & Perry Laboratories (Washington, DC., USA).

**Table 1:** The catalog numbers and dilution factor of the respective antibodies used in this study.

Antibodies	Catalog numbers	Clone name	Dilutionfactor	Manufacture
Mouse anti-M	–	–	2000	Our laboratory
Rabbit anti-p-mTOR (S2448)	2971	ND	1500	Cell Signaling
Rabbit anti-mTOR	2983	7C10	3000	Cell Signaling
Rabbit anti-p-PI3K p85 (Y458)	4228	ND	2000	Cell Signaling
Rabbit anti-PI3K p85	4257	19H8	2000	Cell Signaling
Rabbit anti-p-Akt (T308)	2965	C31E5E	3000	Cell Signaling
Rabbit anti-p-Akt (S473)	3787	736E11	2000	Cell Signaling
Rabbit anti-Akt	2964	5B5	3000	Cell Signaling
Rabbit anti-Atg7	8558	D12B11	3000	Cell Signaling
Rabbit anti-Beclin 1	3495	D40C5	1500	Cell Signaling
Mouse anti-IκBα	4814	L35A5	3000	Cell Signaling
Rabbit anti-p65	4764	C22B4	2000	Cell Signaling
Rabbit anti-p50	3035	ND	2000	Cell Signaling
Rabbit anti-histone H2A	2578	ND	2000	Cell Signaling
Mouse anti-p-Bcl-2 (S70)	O5-613	ND	1500	Upstate
Mouse anti-Bcl-2	15071	124	3000	Cell Signaling
Rabbit anti-p62	7695	D10E10	2000	Cell Signaling
Rabbit anti-p-Src (Y416)	2113	100F9	1500	Cell Signaling
Mouse anti-Src	2110	L4A1	3000	Cell Signaling
Rabbit anti-p-SAPK/JNK (T183/Y185)	9251	ND	2000	Cell Signaling
Rabbit anti-SAPK/JNK	9252	ND	3000	Cell Signaling
Rabbit anti-p-AMPK (T172)	2531	ND	2000	Cell Signaling
Rabbit anti-AMPK	2532	ND	2000	Cell Signaling
Rabbit anti-LC3B	2775	ND	3000	Cell Signaling
Rabbit anti-Cox2	160107	ND	2000	Cayman Chemical
Rabbit anti-EP2	ab167171	ND	2000	Abcam
Rabbit anti-EP4	sc-55596	C-4	1000	Santa Cruz
Mouse anti-β-actin	sc-47778	C4	5000	Santa Cruz

### shRNAs

The shRNAs were constructed using the pGFP-V-RS (TR30007) plasmid from OriGene Co. (Rockville, USA). Based on the results of prelimninary tests, the shRNAs exhibiting the most significant suppression effect to the target gene expression were used in this study. Sequences for shRNAs are as follows: AMPK: CTCCAAGACCAGGAAGTCATACAATAGAA(Cat no: TG505729; Tube ID: GI 505440), EP2: AACTTCCTGTTCTACACAGTCAGATGCCA(Cat no: TG516357; Tube ID: GI340313), EP4: TGGTGCTTCATCGACTGGACCACCAACGT(Cat no: TG516511; Tube ID: GI340311). TurboFect™ *in vitro* transfection reagent (Thermo Fisher Scientific, Waltham, USA) was used for transfection. After 24 h post transfection, cells were infected with BEFV at a multiplicity of infection (MOI) of 1 for further research purposes.

### Cell Viability Assay

Cell viability was determined using the MTT assay to examine for the deleterious effects on cells by the compounds used in this study. MDBK cells were seeded in 4-well plates, grown for 1 day until about 60% confluence, and then treated with the compounds for 24 h. Cells were swirled gently for a few seconds after 50 μl of thiazolyl blue tetrazolium bromide (MTT; Sigma-Aldrich) was added to each well, and then cultured for 3 h. After removing the medium, the cells were washed with PBS twice. 50 μl of supernatant was evaluated at 570 nm for optical density, with subtraction of background at 670 nm.

### Determination of Virus Titer

To explore whether aspirin and AICAR inhibit viral growth, MDBK cells were pretreated with or without aspirin (5 mM) or AICAR (1 mM), respectively, for 30 min and then infected with BEFV at an MOI of 1 for 18 h. The effect of aspirin and AICAR on BEFV production was determined by virus titer. Virus titer was determined as described previously (7). Briefly, BEFV-infected MDBK cell supernatant was collected for determining virus titer by an agar overlay plaque assay carried out in triplicate. Cells in 6-cm cell culture dishes were incubated for 1 h with diluted virus in 100 μl serum-free MEM. The cells were then washed twice with MEM to remove unabsorbed viruses and overlaid with 2 ml of 1% agarose in MEM which contains 5% FBS and antibiotics. Plaques were checked after an incubation period of 2 days at 37°C by staining with neutral red for 3h.

#### Real-Time Quantitative Reverse Transcription and Polymerase Chain Reaction (qRT-PCR)

To investigate whether aspirin and AICAR influence the transcription of the BEFV M gene as well as ATG-related genes of ULK1, Atg7, and LC3, MDCK cells were either drugs-treated or infected with BEFV at an MOI of 1. All cultures were collected and lysed at 18 h post infection (hpi). Total RNA was isolated from drug-treated or virus-infected cells using Trizol and Rneasy Mini Kit (QIAGEN) according to the manufacturer’s protocols. Total RNAs were then subjected to a real-time qRT-PCR as described previously (21). To obtain cDNAs from the RNA samples, reverse transcription was carried out at 42°C for 60 min with 2 μg of total RNA, 4 μl of 2.5 mM dNTP, 500 ng of oligo dT, 5 μl of 5X RT buffer, and 1 μl of M‐MLV reverse transcriptase (200 U/μl) (Promega, Fitchburg, USA), and nuclease‐free water in a total volume of 25 μl. Target cDNAs were further amplified with iQ™ SYBR Green Supermix (Bio-Rad, Hercules, USA) with primers listed in **Table 2**. The reactions contained 0.25 μg total cDNA, 0.5 μl forward and reverse primers (0.5 μM) each, 10 μl of iQ™ SYBR Green Supermix, reagent and PCR grade water to a final volume of 20 μl. The PCR amplification programm was 95°C for 3 min, 35 cycles of 95°C for 15 s, and 56°C for 1 min. Relative quantitation results were analysed with the CFX connect model of real time PCR detection system (Bio-Rad). The glyceraldehyde-3-phosphate dehydrogenase (GAPDH) gene was used as an internal control for normalization.

**Table 2:** Primers used in this study for amplification of the respective targeted genes.

Gene	Accession number	Sequence (5′-3′)	Location	Expectedsize (bp)
BEFV M	AF234533	F: GAGATGGTTACCCTTTTCAAGAAA GG R: TCATGACTTAACTAAGTTAGTGAAACCATG	1–23 672–643	672
ULK1	NM_001205927	F: AAGGGCAGCGCCAGCGAGG R: CGTCCGCCTGGTCCGTGA	2605–2623 3094–3077	490
ATG7	NM_001142967	F: ATGGCCTTTGAGGAACCTTT R: ATGCCTCCCTTCTGGTTCTT	726–745 935–916	210
LC3B	NM_001001169	F: ATGCCGTCCGAGAAAACCTT R: TTACACAGATAATTTCATTCC	1–20 378–358	378
GAPDH	NM-001034034	F: CAAGGTCATCCATGACAACTTTG R: GTCCACCACCCTGTTGCTGTAG	477–499 972–951	496

### PGE2 Assay

MDBK cells were infected with BEFV at an MOI of 1 and cultured for 18 h. The medium was harvested, and the PGE2 concentration in the culture medium of infected MDBK cells was determined using the Prostaglandin E2 EIA Kit (Cayman Chemical Co., Ann Arbor, USA).

### Isolation of Cytoplasmic and Nuclear Protein Fractions

Cellular protein fractions were extracted through serial buffers in the CNM compartmental Protein Extraction Kit (Biochain Institute Inc., Hayward, USA). Cells were suspended in ice-cold buffer C then homogenized by passing through a syringe with a bent 26.5 gauge needle. The supernatant containing cytoplasmic proteins was collected and placed in another tube after centrifugation at 15,000 *g* at 4 °C for 20 min. Cold buffer W was added to wash the pellet then removed after centrifugation at 15,000 *g* at 4°C for 20 min. The pellet was resuspended with cold buffer, centrifuged at 15,000 x*g* at 4 °C for 20 min, and the supernatant containing nuclear proteins was transferred to a clean tube.

### Plasmid Construction

A pH-sensitive GFP-mCherry-LC3 reporter plasmid described previously (7) was used to observe the maturation of autolysosome from autophagosomes. GFP lost its fluorescence under a low pH environment when autophagosomes fuse with lysosomes to form autolysosomes. Thus, GFP-mCherry-LC3 could be a marker to detect autophagosome and autolysosomes.

### Transient Transfection

For the transfection experiments, MDBK cells were seeded into six-well plates. At about 75% confluence, cells were transfected with the respective constructs using Turbofect reagent according to the manufacturer’s instructions (Level Biotechnology Inc., New Taipei City, Taiwan).

### Electrophoresis and Western Blot

Cells were lysed with Laemmli loading buffer (200 mM Tris, pH 6.8; 8% SDS, 10% β-mercaptoethanol, 40% glycerol, 0.04% bromophenol blue) after washing with PBS twice. The cell lysates were collected and boiled for 10 min. Samples were separated on 10% or 15% sodium dodecyl sulphate-polyacrylamide gel electrophoresis (SDS-PAGE) and then transferred to PVDF membranes. Protein expression was detected by using specific primary antibodies and a secondary antibody conjugated with horseradish peroxidase (HRP). The membranes were washed with TBST buffer (50 mM Tris-HCl pH7.5, 150 mM NaCl, and 0.1% Tween 20), soaked with enhanced chemiluminescence solution (ECL plus) (Amersham Biosciences, Little Chalfont, Buckinghamshire, UK), and exposed to X-ray film. Protein band intensity was calculated using the program Photocapt (Vilber Lourmat, France). ImageJ was used to quantify Western blots signals.

### Immunofluorescence Staining

MDBK cells expressing GFP-mCherry-LC3 or GFP-LC3 proteins described previously (7) were seeded on 18 x 18 mm coverslips and cultured for 24 h. The medium was replaced to 2% FBS and the chemical agent of desire for 30 min before BEFV infection. Cells were infected with BEFV at an MOI of 1 and fixed with 4% paraformaldehyde in PBS for 1 h at room temperature, followed by soaking in PBS with 0.3% Triton X-100 for 10 min. After washing with PBS, the cells were blocked with SuperBlock^®^ T20 (PBS) blocking buffer (Thermo Fisher Scientific, Waltham, USA) at 4°C for 30 min. Cell nuclei were stained with 4’, 6-diamidino-2-phenylindole (DAPI) for 10 min in the dark, followed by observation with a BX51 fluorescence microscope (Olympus,Tokyo, Japan). The coverslips were washed with PBS three times at room temperature and then mounted onto glass slides using ibidi mounting medium (ibidi GmbH, Lochhamer Schlag, Germany). The LC3 punta images were observed under fluorescence microscopy and the montage was edited using Adobe Photoshop CC.

### Statistical Analysis

All data obtained in this study were evaluated for statistical significance according to student t-test or the Duncan’s multiple range test (DMRT) using SPSS software (version 20.0). P values that were less than 0.05 were considered statistically significant (22).

## Results

### Aspirin and AICAR Transcriptionally Downregulate the BEFV M Gene and Inhibit Virus Replication

**Figure 1:**
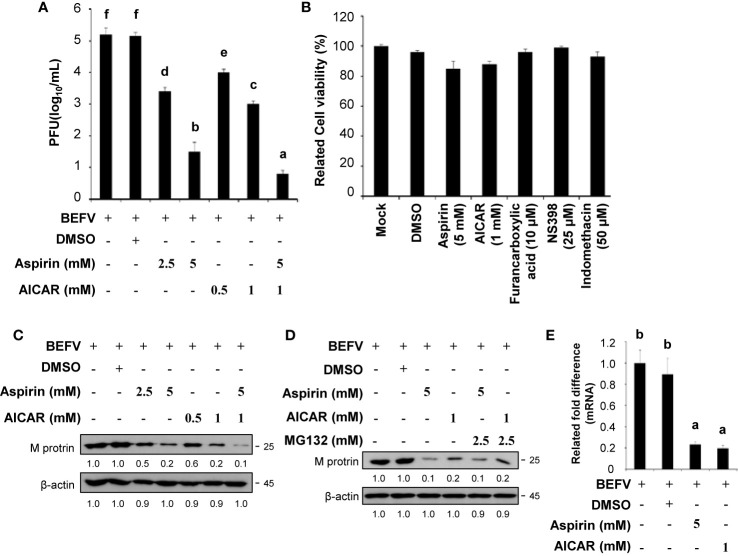
Aspirin and 5-aminoimidazole-4-carboxamide-1-β-riboside (AICAR) inhibit viral growth. **(A)** Madin-Darby bovine kidney (MDBK) cells were pretreated with or without aspirin (2.5 and 5 mM) or AICAR (0.5 and 1 mM), respectively, for 30 min and then infected with BEFV at an MOI of 1 for 18 h. The effect of aspirin and AICAR on BEFV production was determined. Significance between the treatments was determined by Duncan’s Multiple Range Test (MDRT) using SPSS software (Version 20.0). Means with common alphabets (a, b, c, d,e, f) denotes no significance at p <0.05. Each value represents mean ± SE of three independent experiments. **(B)** To examine whether the compounds used in this study had the deleterious effects on cells, cell viability was determined using the MTT assay. Each value represents mean ± SE of three independent experiments. **(C, D)** The levels of the BEFV M protein in aspirin- and AICAR-treated MDBK cells were examined **(C)** in the presence or absence of proteasome inhibitor MG132 **(D)**. The levels of indicated proteins in the BEFV-infected group were considered onefold. The inactivation folds indicated below each lane were normalized against values for the BEFV-infected group. Protein levels were normalized to those for β‐actin. Signals in all Western blots were quantified with ImageJ software. All experiments were conducted in three independent experiments. **(E)** The BEFV M and GADPH mRNA levels were quantified by real-time qRT-PCR in BEFV-infected MDBK cells in the presence or absence of indicated drugs. In real-time qRT-PCR amplification of the M and GADPH genes, MDBK cells were infected with BEFV at an MOI of 1. The BEFV-infected cells were collected at either 24 hpi, and total RNAs were extracted for real-time qRT-PCR. Significance between the treatments was determined by Duncan’s Multiple Range Test (MDRT) using SPSS software (Version 20.0). Means with common alphabets (a, b) denotes no significance at p <0.05. Each value represents mean ± SE of three independent experiments.

As reported previously, we found that Cox-2 and PGE2 are important in BEFV entry and subsequent replication (12). More recently, we demonstrated that BEFV-induced autophagy enhancing virus replication *via* upregulation of the Src/JNK/AP1 and PI3K/Akt/NF-κB pathways and suppression of the PI3K/Akt/mTOR pathway (7). In this study, we further exploreed whether aspirin and AICAR affect Cox-2 and the signaling pathways, which are involved in virus entry and induction of autophagy. The concentrations that produced a 50% inhibitory effect (IC50) of aspirin and AICAR have been shown previously (23, 24). MDBK cells were infected with BEFV at an MOI of 1 for 18 h with or without pretreatment of two comcentions of aspirin and AICAR, respectively. In this work,virus yield was significantly reduced in aspirin- and AICAR-treated MDBK cells (**Figure 1A**), suggesting that these drugs have a potential anti-viral ability. To examine whether all compounds used in this study had the deleterious effects on MDBK cells, **c**ell viability was determined using the MTT assay. As shown in **Figure 1B**, cell viability was slighly reduced compared to the cases with mock treatments. Recently, we have shown that the BEFV M protein is one of major protein that is involved in BEFV-induced autophagy (7). Thus, the levels of the BEFV M protein were also analyzed in aspirin- and AICAR-treated MDBK cells. Our results reveal tha both aspirin and AICAR reduced the levels of BEFV M protein (**Figure 1C**). Furthermore, in the presence of proteasome inhibitor MG132, the decreased level of M protein was not reversed in aspirin- and AICAR-treated MDBK cells (**Figure 1D**), suggesting that these drugs reeduce the level of the BEFV M protein is independent of the proteaseome pathway. To further examine whether aspirin or AICAR transcriptionally downregulate the BEFV M, the M mRNA levels in aspirin- and AICAR-treated MDBK cells were examined. As shown in **Figure 1E**, the M mRNA levels in aspirin- and AICAR-treated MDBK cells were reduced as comparsion to those in untreated cells, suggesting that the BEFV M is transcriptionally downregulated by aspirin and AICAR.

### Cox-2 Is Essential for BEFV-Induced Autophagy

**Figure 2:**
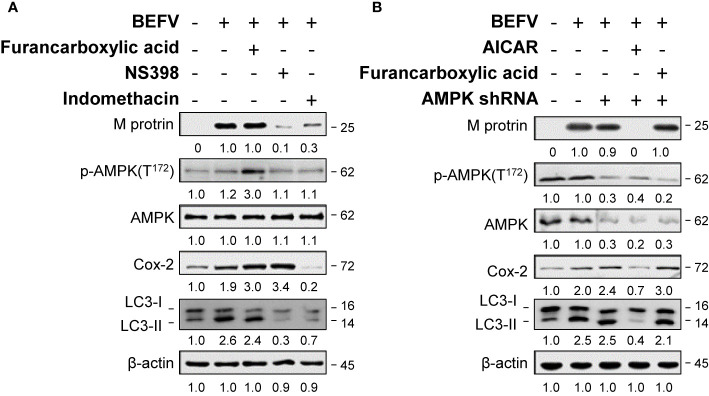
BEFV-induced autophagy requires Cox-2 and is AMPK-independent.** (A)** MDBK cells were pretreated either with Furancarboxylic acid, NS-398 (25 μM) and indomethacin for 1 h followed by infection with BEFV at an MOI of 1 for 18 h. **(B)** MDBK cells were transfected with AMPK shRNA for 6 h, then with or without pretreatment of Furancarboxylic acid or AICAR for 1 h, followed by infection with BEFV at an MOI of 1. The cell lysates were harvested at 18 hpi and subjected to immunoblotting using antibodies as indicated. The levels of indicated proteins in the mock group were considered onefold. The activation and inactivation folds indicated below each lane were normalized against values for the mock control group. Protein levels were normalized to those for β‐actin. Signals in all Western blots were quantified with ImageJ software. All experiments were conducted in three independent experiments. The predicted size of each protein was labeled at the right-hand side in kDa.

We have shown previously that BEFV up-regulates the expression of Cox-2 in a time-dependent manner (12). Cox-1 and Cox-2 are two isozymes of cyclooxygenase which are encoded by individual genes and exert functional differences (25). It is interesting to study the roles of each after BEFV entry. As illustrated in **Figure 2A**, the levels of Cox-2 and LC3-II increased in BEFV-infected MDBK cells. Selective inhibition of the activity of Cox-2 by NS398 (26) reduced the levels of the BEFV M protein and LC3-II while the Cox-2 level increased (**Figure 2A**). Our results were consistent with previous studies that suggest NS-398 blocks Cox-2 activity but upregulates Cox-2 (27–29). Indomethacin is one of the NSAIDs, which non-selectively inhibits cyclooxygenase activity but is more potent against Cox-1 than Cox-2 (30). A concomitant decreased expression level of Cox-2 by indomethacin was also reported (31–33). Although an increased level of Cox-2 induced by BEFV was notably reduced by indomethacin, the decrease in the levels of the BEFV M protein and LC3-II were less pronounced as compared to NS398 (**Figure 2A**). Collectively, our findings reveal that inhibition of Cox-2 reduces the levels of the BEFV M protein and LC3-II.

### Suppression of BEFV-Induced Autophagy by AICAR Is Independent of AMPK

AMPK is a serine-threonine kinase up-regulated by various stimuli through sensing an elevated intracellular AMP/ATP ratio. AMPK signaling is involved in multiple metabolic reprogramming and cell growth as well as autophagy. Accordingly, we investigated the role of AMPK in BEFV-induced autophagy. The effect of AMPK on BEFV was examined by measuring the expression level of the BEFV M protein. As shown in **Figure 2A**, the increased levels of Cox-2 and LC3-II were seen in BEFV-infected MDBK cells. Activation of AMPK by an AMPK activator, furancarboxylic acid, increased the phosphorylated forms of AMPK (p-AMPK) and Cox-2 level in BEFV-infected cells (**Figure 2A**). Interestingly, elevated levels of Cox-2 and LC3-II in BEFV-infected or furancarboxylic acid pre-treated groups were not altered in AMPK-knockdown cells (**Figure 2B**). Collectively, these data reveal that BEFV-induced autophagy is not regulated by AMPK signaling. In contrast, AICAR as an AMPK activator reduced the levels of BEFV M protein, Cox-2, LC3-II in BEFV-infected cells, and AMPK-knockdown cells (**Figure 2B**). Our finding suggests that AICAR inhibits BEFV-induced autophagy through an AMPK-independent mechanism.

### Aspirin and AICAR-Mediated Inhibition of BEFV-Activated Prostaglandin E2 (PGE2) and G-Protein-Coupled E-Prostanoid Receptors 2 (EP2)

**Figure 3:**
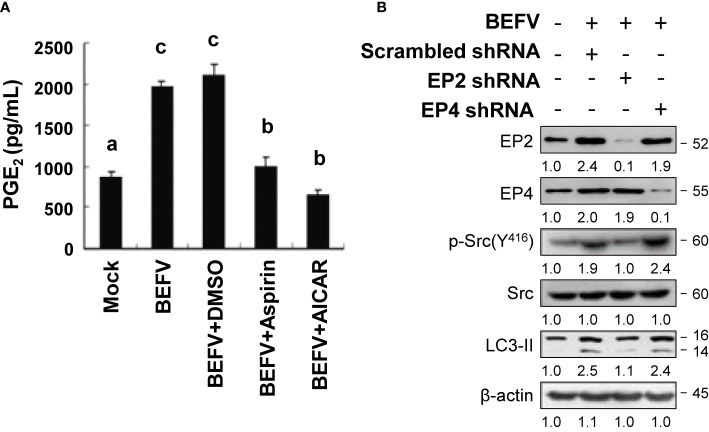
BEFV triggers the PGE2/EP receptor pathway. **(A)** MDBK cells were pretreated with or without aspirin (5 mM) or AICAR (1 mM), respectively, for 30 min and then infected with BEFV at an MOI of 1 for 18 h. Supernatants of BEFV-infected cells were harvested for quantification of PGE2 measured by enzyme linked immunosorbent assay (ELISA). Significance between the treatments was determined by Duncan’s Multiple Range Test (MDRT) using SPSS software (Version 20.0). Means with common alphabets (a, b, c) denotes no significance at p <0.05. Each value represents mean ± SE of three independent experiments. **(B)** MDBK cells were transfected with scrambled (negative control), EP2, and EP4 shRNAs, respectively, for 24 h, followed by infection with BEFV at an MOI of 1 for 18 h. The effect of E-prostanoids (EPs) on BEFV-induced LC3 II was analyzed by immunoblotting. The levels of indicated proteins in the mock group were considered onefold. The activation and inactivation folds indicated below each lane were normalized against values for the mock control group. β‐actin was used for the internal control for normalization. Signals in all Western blots were quantified with ImageJ software. All experiments were conducted in three independent experiments. The predicted size of each protein was labeled at the right-hand side in kDa.

Our earlier study has shown that BEFV activates the Src/JNK/AP1 and PI3K/Akt/NF-κB pathways to induce Cox-2-mediated production of intracellular PGE2 at the stage of virus entry (12). PGE2 interacts with EP2 and EP4, further enhancing the Src/JNK/AP1 and PI3K/Akt/NF-κB pathways in an autocrine or paracrine fashion to increase virus entry (12). More recently, we also found that BEFV triggers autophagy *via* activation of the Src/JNK/AP1 pathway (7). The crosstalk between PGE2 and autophagy induction and the potential role of EP-mediated signaling in BEFV-induced autophagy were further investigated. In this work, MDBK cells were infected with BEFV at an MOI of 1 for 18 h, with or without pretreatment of AICAR or aspirin, respectively. As shown in **Figure 3A**, BEFV-induced accumulation of PGE2 was observed. Both aspirin and AICAR reduced the concentrations of PGE2 elevated by BEFV. We next analyzed the effect of EPs on BEFV-induced autophagy. We found that knockdown of EP2 *via* a specific shRNA reversed the increased levels of p-Src and LC3-II by BEFV while there is no obvious inhibitory effect on EP4 knockdown cells (**Figure 3B**). These results suggested that activation of a PGE2/EP2 signal to amplify the Src/JNK/AP1 pathway is important for BEFV to induce autophagy.

### Reversion of the BEFV-Activated PI3K/Akt/NF-κB and Src/JNK/AP1 Pathways as Well as the BEFV-Anactivated PI3K/Akt/mTOR Pathway by AICAR

**Figure 4:**
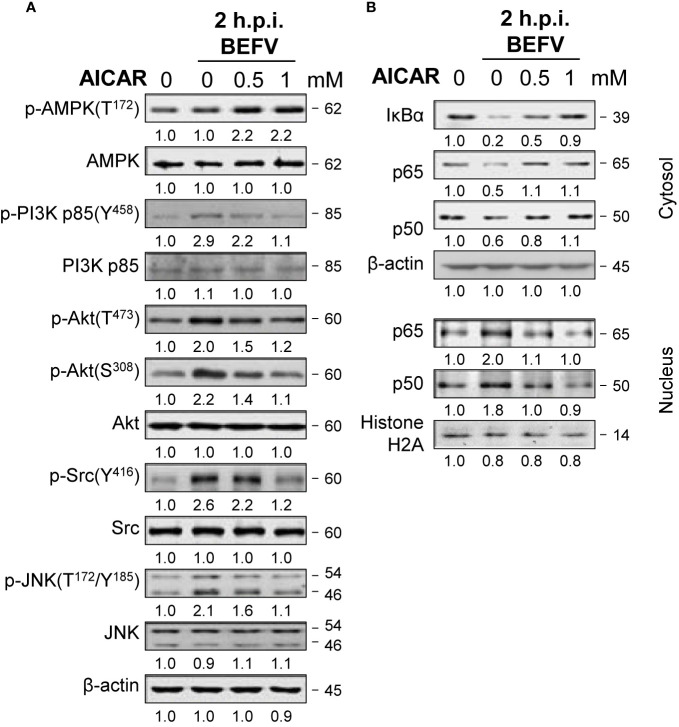
BEFV activates the PI3K/Akt/NF-κB and Src/JNK/AP-1 pathways in the early stage and is suppressed by 5-aminoimidazole-4-carboxamide-1-β-riboside (AICAR). MDBK cells were pretreated with or without AICAR (0.5 and 1 mM), respectively, for 30 min, followed by infection with BEFV at an MOI of 1 for 2 h. **(A)** The cell lysate or **(B)** cytosolic and nuclear fraction were collected and subjected to immunoblotting with the respective antibodies as indicated. The levels of indicated proteins in the mock group were considered onefold. The activation and inactivation folds indicated below each lane were normalized against values for the mock control group. β‐actin was used for the internal control for normalization.Signals in all Western blots were quantified with ImageJ software. All experiments were conducted in three independent experiments. The predicted size of each protein was labeled at the right-hand side in kDa.

**Figure 5:**
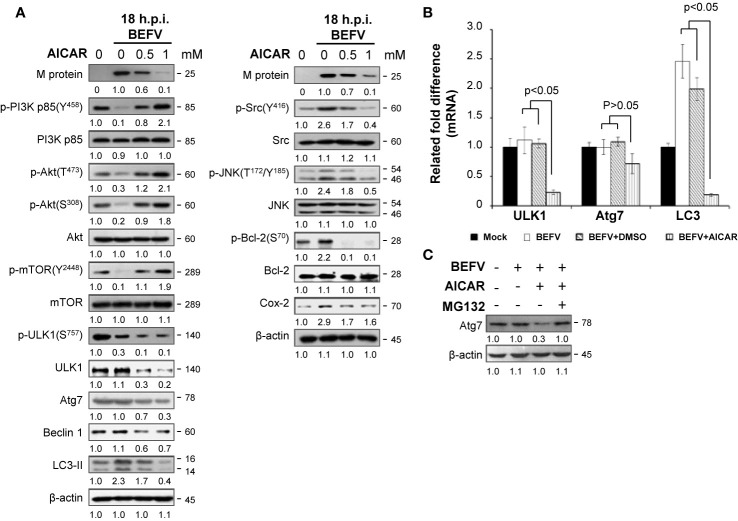
BEFV induces downregulation of the PI3K/Akt/mTOR pathway and autophagy in the late stage, suppressed by 5-aminoimidazole-4-carboxamide-1-β-riboside (AICAR). **(A)** MDBK cells were pretreated with or without AICAR (0.5 and 1 mM), respectively, for 30 min, followed by infection with BEFV at an MOI of 1 for 18 h. The cell lysate was collected and subjected to immunoblotting with the respective antibodies as indicated. **(B)** Total cellular RNA of BEFV-infected MDBK cells was extracted, transcribed into cDNA, and subjected to real-time qRT-PCR. The mRNA levels of ULK1, Atg7, and LC3 were normalized to that of GAPDH. The mRNA levels in mock-treated cells was considered 100%. All data obtained in this study were analyzed by student t-test and expressed as averages of three independent experiments. P values of less than 0.05 were considered significant. **(C)** MDBK cells were pretreated with AICAR alone or in combination with MG132 (5 mM) for 30 min, followed by infection with BEFV at an MOI of 1 for 18 h. Cell lysates were collected and subjected to immunoblotting with the antibody specific to Atg7. The levels of indicated proteins in the mock group **(A, C)** were considered onefold. The activation and inactivation folds indicated below each lane were normalized against values for the mock control group. Protein levels were normalized to those for β‐actin. Signals in all Western blots were quantified with ImageJ software. All experiments were conducted in three independent experiments. The predicted size of each protein was labeled at the right-hand side in kDa.

Having shown that AICAR inhibits BEFV-induced autophagy through an AMPK-independent manner. We next wanted to explore the mechanisms of AICAR on suppression of BEFV-induced autophagy. As shown in **Figure 4A**, the BEFV-induced increased levels of the phosphorylated form of PI3Kp85 (Y458), Akt (T308 and S473), Src (Y416), and JNK(T183/T185) were reversed by AICAR in a dose-dependent manner. BEFV-modulated inhibition of IκBα and nuclear translocation of NF-κB subunits (p50 and p65) were simultaneously reversed by AICAR in a dose-dependent manner (**Figure 4B**). These results suggest that the PI3K/Akt/NF-κB and Src/JNK/AP1 pathways activated at an early stage of BEFV infection were reversed by AICAR. At the late stage of BEFV infection, the expression level of the BEFV M protein was dramatically reduced in AICAR-treated cells (**Figure 5A**). Recently, we showed that the BEFV M protein suppresses the PI3K/Akt/mTORC1 pathway to enhance BEFV-induced autophagy (7). In the current study, BEFV-induced decreased levels of p-PI3Kp85 and p-Akt along with p-mTOR were not further suppressed but were increased by AICAR treatment (**Figure 5A**). All of these reversions induced by AICAR were dose-dependent (**Figures 4A, B**, **5A**).

Having demonstrated that multiple pathways regulated by BEFV were reversed by AICAR, we further investigated the effect of AICAR to Atg-related protein expression in BEFV-infected cells. Autophagy consists of several sequential steps including induction, autophagosome formation, degradation and reuse (34). Class III phosphatidylinositol 3-kinase complex I (PI3KC3-C1) and the ULK1 complex are two major initiation complexes involving in commencement of autophagy. Beclin 1 is a constituent of the PI3KC3-C1 complex, and Bcl-2 binds to Beclin 1 to inhibit autophagy (35). Phosphorylation of Bcl-2 to dissociate Beclin 1 can be induced by activating JNK to initiate autophagy (36). We have shown that BEFV increases JNK-mediated phosphorylation of Bcl-2 and the level of Cox-2 for autophagy (7). The present study reveals that increased levels of Bcl-2 phosphorylation induced by the Src-JNK pathway and the increased level of Cox-2 induced by BEFV were reversed by AICAR (**Figure 5A**). Phosphorylation of Beclin 1 by ULK1 is required for full autophagy induction (36). Suppression of the PI3K/Akt signal decreases phosphorylation of mTOR at Ser^2448^ and results in inactivation of mTOR, which hence loses its ability to control ULK1 by phosphorylation at site S^757^ so as to activate ULK1. As illustrated in **Figure 5A**, BEFV suppressed the PI3K/Akt/mTOR pathway to reduce phosphorylation of ULK1 (S^757^), but AICAR did not reverse this and the level of p-ULK1 was even further decreased (**Figure 5A**). We also observed that AICAR decreases the level of Atg-related proteins including ULK1, Atg7, and Beclin 1 (**Figure 5A**). To investigate the precise mechanism of AICAR on these ATG-related proteins, real-time qRT-PCR was carried out. Data presented in **Figure 5B** demonstrate a suppression effect of AICAR on mRNA expression of the ULK1 and LC3 genes except for Atg7, suggesting that AICAR transcriptionally downregultes ULK1 and LC3. Furthermore, co-treatment of AICAR with MG132 counteracted the effect of AICAR on the reduction of Atg7 levels (**Figure 5C**), suggesting that AICAR enhances Atg7 degradation by the proteasome pathway. Collectively, our results reveal that AICAR suppresses BEF-induced autophagy *via* suppression of ATG-related proteins of ULK1, Atg7, and LC3.

### Reversion of the BEFV-Activated PI3K/Akt/NF-κB and Src/JNK/AP1 Pathways in the Early Stage of Infection and the BEFV-Suppressed PI3K/Akt/mTOR Pathway in the Late Stage of Infection by Aspirin

**Figure 6:**
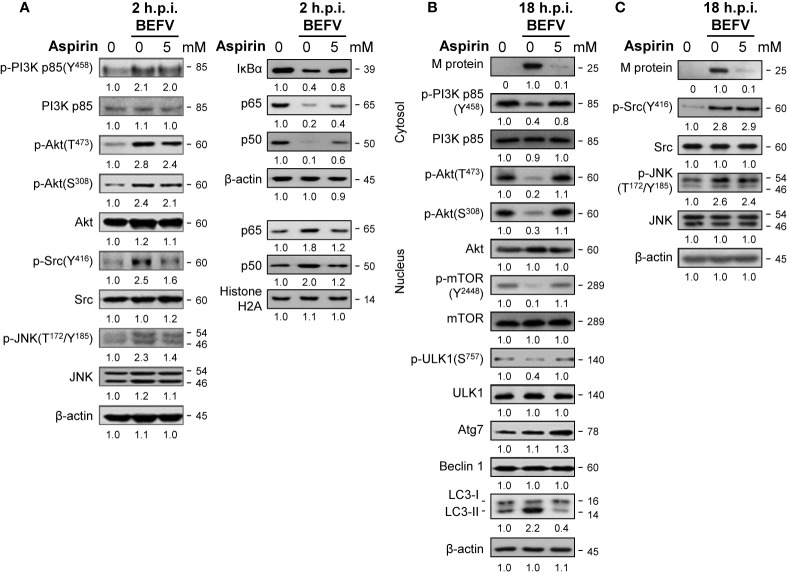
Aspirin suppresses the BEFV-activated Src/JNK/AP-1 pathway at the early stage and reverses the BEFV-inactivated PI3K/Akt/mTOR pathway at late stage. MDBK cells were pretreated with or without aspirin (5 mM), respectively for 30 min, followed by infection with BEFV at an MOI of 1 for 2 hpi **(A)** and 18 hpi **(B, C)**. The cell lysates or cytosolic and nuclear fraction were collected at the indicated time points and subjected to immunoblotting with the respective antibodies. The levels of indicated proteins in the mock control group were considered onefold. The activation and inactivation folds indicated below each lane were normalized against values for the mock control group. Protein levels were normalized to those for β‐actin. Signals in all Western blots were quantified with ImageJ software. All experiments were conducted in three independent experiments. The predicted size of each protein was labeled at the right-hand side in kDa.

Having shown that aspirin significantly inhibites virus yield (**Figure 1A**), we next wanted to explore the precise mechanism of the suppression effect of aspirin on BEFV replication. MDBK cells were pretreated with or without aspirin (5 mM) for 30 min followed by infection with BEFV at an MOI of 1. Cell lysates were collected and immunoblotted with the respective antibodies. As illustrated in **Figure 6A**, at the early stage of BEFV infection, increased levels of p-Src and p-JNK induced by BEFV were moderately reversed by aspirin while increased levels of p-PI3K and p-Akt induced by BEFV were only slightly reversed by aspirin (**Figure 6A**). However, degradation of IκBα and nuclear translocation of NF-κB subunits (p50 and p65) were reversed by aspirin (**Figure 6A**). At the late stage of BEFV infection, the decreased levels of p-PI3Kp85, p-Akt, p-mTOR, and p-ULK1 were detected and reversed by aspirin while increased levels of p-Src and p-JNK induced by BEFV were not reversed by aspirin (**Figure 6C**). Similar to AICAR treatment, the expression level of the BEFV M protein was dramatically reduced in aspirin-treated cells at the late stage of BEFV infection (**Figures 6B, C**). The BEFV-induced increased level of LC3-II was dramatically reversed by aspirin. In contrast to AICAR, the levels of Atg7, Beclin 1, and ULK were not affected in aspirin-treated cells (**Figures 5A**, **6B**). Collectively, these results show that aspirin reverses BEFV-mediated degradation of IκBα, upregulates PI3K/Akt/NF-κB and Src/JNK/AP1 pathways in the early stage of infection, and downregulates the PI3K/Akt/mTOR pathway in the late stage of infection. Our findings suggest that aspirin inhibits BEFV-induced autophagy, thereby inhibiting virus propagation.

### Reversion of Autophagy Flux Delayed by BEFV by Aspirin and AICAR

**Figure 7:**
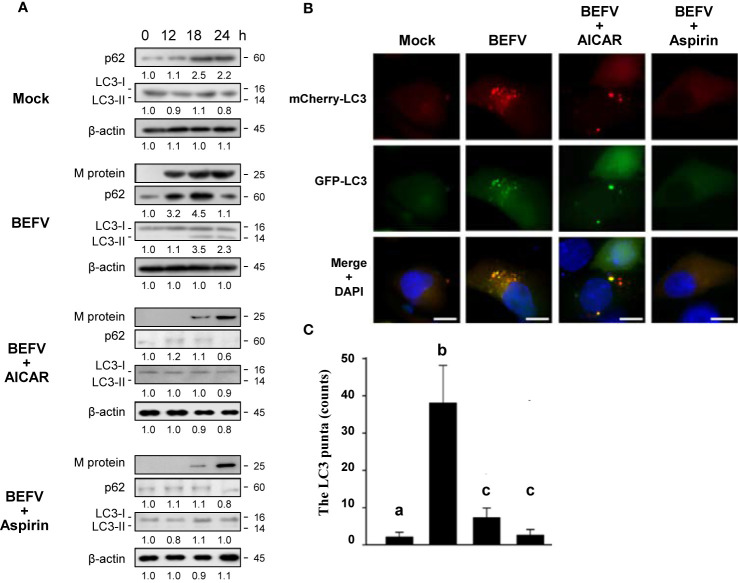
Delayed autophagosome formation by BEFV is suppressed by 5-aminoimidazole-4-carboxamide-1-β-riboside (AICAR) or aspirin. **(A)** MDBK cells were infected with BEFV at an MOI of 1 with or without pretreatment of AICAR (1 mM) and aspirin (5 mM), respectively. Cell lysates were collected at the indicated time points and subjected to immunoblotting with the respective antibodies. The levels of indicated proteins in the mock group were considered onefold. The activation and inactivation folds indicated below each lane were normalized against values for the mock control group. β‐actin was used for the internal control for normalization. All experiments were conducted in three independent experiments. Signals in all Western blots were quantified with ImageJ software. The predicted size of each protein was labeled at the right-hand side in kDa. **(B)** Induction of autophagy in MDBK cells expressing the mCherry-GFP-LC3 protein was monitored after pretreatment with AICAR or aspirin, respectively, for 30 min and then infected with BEFV at an MOI of 1 for 18 h. LC3 fluorescent puncta were observed using fluorescence microscopy. Cell nuclei were stained with DAPI. Scale bars, 25 mm. **(C)** The numbers of LC3 punta were calculated from the results from panel **(B)**. Significance between the treatments was determined by Duncan’s Multiple Range Test (MDRT) using SPSS software (Version 20.0). Means with common alphabets (a, b, c) denotes no significance at p <0.05. Each value represents mean ± SE of three independent experiments.

The final process of autophagy is degradation and reused by fusion of autophagosomes with lysosomes to form autolysosomes. Our recent report showed that autophagic flux was delayed during BEFV infection (7). It is interesting to investigate whether aspirin or AICAR affect the autophagic flux. BEFV-infected MDBK cells were pretreated with or without AICAR (1 mM) and aspirin (5 mM), respectively. Cell lysates were collected at the indicated time points and the levels of M protein and autophagic protein markers including p62 and LC3-II were analyzed. p62 is a multifunctional protein and serves as an autophagic flux reporter and integration center for the autophagosome and ubiquitin-proteasome system (37). It interacts with LC3-II and is selectively degraded by the autophage-lysosome pathway. As shown in **Figure 7A**, the increased level of p62 was further increased at 12 hpi and then decreased at 24 hpi., suggesting that BEFV protects p62 from degradation *via* an unknown mechanism before completing virus replication. The increased level of lipid conjugated LC3-II by BEFV became evident at 18 hpi. and then decreased at 24 hpi. The expression level of M protein was decreased in AICAR- or aspirin-treated MDBK cells as compared to BEFV infection alone (**Figure 7A**). This is consistent with the data shown in **Figure 1**. Pretreatment with AICAR or aspirin, respectively, inhibited the accumulation of p62 or LC3-II induced by BEFV (**Figure 7A**). To further confirm this observation, we used a pH-sensitive GFP-mCherry-LC3 reporter plasmid to examine the maturation process of autophagosomes. GFP-LC3 is unstable in the lysosomal acidic and degradative conditions, while mCherry-LC3 is relative stable (7). After 18 hpi., the puncta of cells pretreated with AICAR or aspirin were significantly reduced (**Figure 7B**), suggesting that autophagosome formation was inhibited by aspirin and AICAR. The count of GFP-mCherry-LC3 puncta was significantly diminished in drug pretreated cells as compared to BEFV-infected cells (**Figure 7C**). These results indicate that delayed autophagy flux induced by BEFV was inhibited by aspirin or AICAR.

## Discussion

The present study provides a promising approach to inhibit BEFV replication for therapeutic purposes. We demonstrate for the first time that AICAR and aspirin attenuate BEFV replication by inhibiting BEFV-induced autophagy *via* suppression of the BEFV-activated PI3K/Akt/NF-κB and Src/JNK pathways and reversion of the BEFV-suppressed PI3K/Akt/mTOR pathway. Our results also reveal that aspirin and AICAR negatively regulate the BEFV M protein, which is one of the major protein for BEFV-indiced autophagy (7). Autophagy is the cellular catabolic process in which cytoplasmic target material is transported to lysosomes for degradation *via* phagosomes, which are a double-membrane vacuoles (38). This is a self-degradative process in response to nutrient stress or balancing sources of energy at critical conditions (39) and acts as a cell-intrinsic anti-viral immune defense (40). Autophagy plays a role in immunological processes to direct elimination of microbes, control inflammation, and also plays a role in antigen presentation, lymphocyte homeostasis, and secretion of immune mediators (41). Although viruses can be eliminated through the innate immune response and selective degradation of immune components with viral particles by autophagy, viruses develop diverse strategies such as evasion of autophagic degradation, manipulation of autophagosomes, regulation of lipophagy, and exocytosis to hijack and subvert autophagy for their replication (42–46). Autophagy can be induced by suppression of mTOR or activation of AMPK. Our recent study demonstrated that BEFV triggers autophagy to beneft its replication through suppression of the PI3K/Akt/mTOR pathway (12). AMPK serves as a double-edged sword to viruses and its activation is essential for some viruses. For example, the p17 protein of avian reovirus activates AMPK to induce autophagy for replication (42), Bluetongue virus induces autophagy through activation of AMPK to sustain viral replication (47), and respiratory syncytial virus induces autophagy through ROS and AMPK activation, which is beneficial for viral replication (48). On the other hand, activation of AMPK is lethal to certain viruses such as hepatitis C virus (HCV) (49), Epstein-Barr virus (50), and herpes simplex virus (51). AICAR is a common AMPK activator. Several studies suggested that AICAR activates AMPK activity, resulting in inhibition of HCV (52), herpes simplex virus type 1 (53), Coxsackievirus B3 (54), Kaposi’s sarcoma-associated herpes virus (55), and hepatitis B virus (HBV) (56). In the current study, AICAR suppresses BEFV replication by inhibiting autophagy. However, autophagy induced by BEFV is AMPK independent. Our findings presented in this work revealed that AICAR inhibits BEFV-induced autophagy is in an AMPK-independent manner. AICAR does not directly activate AMPK but is metabolized to a direct activator, ZMP (57). ZMP as an AMP mimetic is an intermediate for *de novo* purine biosynthesis of inosine monophosphate (IMP) (20). ZMP binds to and activates a riboswitch to directly regulate the expression of one-carbon metabolism genes in multiple bacterial lineages (22), and is regarded as a master regulator of one-carbon metabolism (58). It should be taken into account that AICAR is able to activate many other AMP-dependent enzymes, such as fructose-1, 6-bisphosphatase (59). Several studies have shown that cellular metabolism regulated by AICAR is AMPK-independent, including apoptosis induction in Jurkat cells (60), inhibition of glucose phosphorylation in rat hepatocytes (61), induction of nuclear translocation of Nrf2 in hepatocytes (62), suppression of LPS-induced iNOS & Cox-2 mRNA/protein (63), decreased transcription of NF-κB-dependent genes (23), and inhibitory inflammatory responses in macrophages (64).

Prostaglandin E2 is prostanoid that was first discovered in 1964 (65). Synthesis of prostanoids through eicosanoid metabolism is initiated from hydrolysis of plasma membrane phospholipid to arachidonic acid by phospholipase A2. Arachidonic acid is converted to PGH2 *via* cyclooxygenase (Cox-1/Cox-2). Prostaglandin E2 is isomerized from PGH2 by tissue specific prostaglandin E synthases (66, 67). PGE2 binds to its corresponding receptors (EP1-4) to act on cells with a wide variety of effects. By modulating inflammation and the immune system through regulating cytokines, the influence on viral infection is virus-family-dependent (68). Our previous studies suggested that BEFV stimulates the Cox-2-mediated PGE2/EP receptor signalling pathways to amplify the Src/JNK pathway for cell entry and autophagy induction (7, 12). In the present study, we further found that AICAR reversed BEFV-mediated increased Cox-2 expression and PGE2 production, thereby inhibiting autophagy and virus yield. Suppression of Cox-2 expression by AICAR has been reported (63, 69, 70). Our findings are consistent with these studies. Aspirin blocks the function of Cox-2 to decrease PEG2 production. Reduction of BEFV-triggered PGE2 production by both AICAR and aspirin is through different mechanisms.

**Figure 8:**
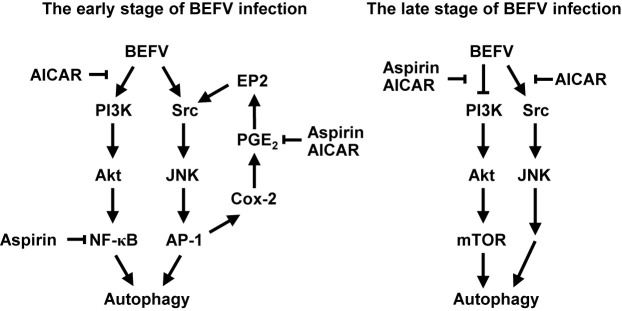
Models illustrating the mechanisms of AICAR and aspirin-mediated inhibition of BEFV-induced autophagy. At the early stage of BEFV infection, AICAR suppresses the BEFV-activated PI3K/Akt/NF-κB and Src/JNK/AP-1/Cox-2/PGE2/EP2 pathways, and aspirin suppresses NF-κB and the BEFV-activated Src/JNK/AP-1/Cox-2/PGE2/EP2 pathway. At the late stage of BEFV infection, AICAR reverses the BEFV-inactivated PI3K/Akt/mTOR pathway and the BEFV-activated Src/JNK/Bcl-2 pathway while aspirin reverses the BEFV-inactivated PI3K/Akt/mTOR pathway.

At the early stage of infection, both Src/JNK/AP-1 and PI3K/Akt/NF-κB pathways are up-regulated to induce autophagy. As illustrated in **Figure 8**, AICAR reverses the BEFV-activated signaling pathways at the early and late stages of infection in a AMPK-independent manner. Furthermore, the ATG-related proteins including ULK1, Beclin 1, ATG7, and LC3 were all suppressed in AICAR-treated but not in aspirin-treated cells. At the late stage of infection, AICAR reversed the BEFV-activated Src/JNK/AP-1 and BEFV-suppressed PI3K/Akt/mTORC1 pathways, but aspirin did not regulate the Src/JNK/AP-1 pathway. Although the BEFV-activated PI3K/Akt pathway at the early stage of infection was not suppressed by aspirin, there was still moderate suppression of NF-κB by aspirin. The inhibition is granted by the innate ability of aspirin, since salicylate inhibition of the NF-κB pathway has been well recognized for a decade (71, 72).

Aspirin is a widely and historically used medication. It is utilized for analgesic, anti-inflammation and anti-thrombosis properties due to inhibition of Cox-1 and Cox-2 activity. Recent studies suggested that aspirin serves as a chemopreventive agent according to the Cox-independent mechanisms including inhibition of NF-κB, interruption of extracellular signal-regulated kinases (ERK), induction of apoptosis by caspase activation and inhibition of the Wnt/b-catenin pathway (73). Thus, the possible applications of aspirin are more than just as a Cox inhibitor. Several studies revealed that aspirin induces autophagy in murine hepatocarcinoma, sarcoma (74), and colorectal cancer cells (75), and inhibits histone acetyltransferase (EP300) to induce autophagy (76). Conversely, aspirin may inhibit autophagy in epithelial cells of the gastrointestinal tract (77) and alleviates cardiac fibrosis in mice by inhibiting autophagy (78). The antiviral ability of aspirin has been previously reported, including influenza (79, 80), cytomegalovirus (81), RNA viruses of the respiratory tract (82), feline foamy virus (83), and Zika Virus (84); however, the underlying mechanisms remain largely unknown. Our study demonstrats that aspirin inhibits BEFV replication by inhibiting BEFV-induced autophagy *via* suppression of the BEFV-activated Src/JNK/AP-1 pathway and its NF-κB-inhibiting activity and revision of the BEFV-suppressed PI3K/Akt/mTORC1 pathway.

The current study provides novel insights into aspirin- and AICAR-impeded virus replication by inhibiting BEFV-induced autophagy *via* suppression of the PI3K/Akt/NF-κB and Src/JNK pathways as well as reversion of the BEFV-inactivated PI3K/Akt/mTORC1 pathway. Hypothesized models for suppression of BEFV-induced autophagy by aspirin and AICAR are shown in **Figure 8**. The present study provides an option for treatment of BEF by aspirin and AICAR.

## Data Availability Statement

The original contributions presented in the study are included in the article. Further inquiries can be directed to the corresponding authors.

## Author Contributions

H-HT, W-RH, C-YC, and H-CC performed most of the experiments. H-HT, W-RH, B-LN, T-LL and H-JL analyzed the data. H-JL conceived and designed the experiments, wrote the paper, and supervised the project. H-JL and B-LN revised and edited the manuscript. All authors contributed to the article and approved the submitted version.

## Funding

This work was financially supported by the Ministry of Science and Technology of Taiwan (MOST 109-2313-B-005-006-MY3), the iEGG and Animal Biotechnology Center from The Feature Areas Research Center Program within the framework of the Higher Education Sprout Project by the Ministry of Education (MOE) in Taiwan (109S0023A), the Taichung Veterans General Hospital (TCVGH-NCHU1097602), and the National Chung Hsing University (TCVGH-NCHU1097602).

## Conflict of Interest

The authors declare that the research was conducted in the absence of any commercial or financial relationships that could be construed as a potential conflict of interest.
